# The P2X7 Receptor in Tumor Immunity

**DOI:** 10.3389/fcell.2021.694831

**Published:** 2021-06-22

**Authors:** Fabio Grassi, Benedetta De Ponte Conti

**Affiliations:** ^1^Institute for Research in Biomedicine, Faculty of Biomedical Sciences, Università della Svizzera Italiana, Bellinzona, Switzerland; ^2^Graduate School for Cellular and Biomedical Sciences, University of Bern, Bern, Switzerland

**Keywords:** P2X7, extracellular ATP, tumor, innate immunity, adaptive immunity

## Abstract

Extracellular adenosine triphosphate (eATP) is a potent mediator of the immune response via stimulation of purinergic P2 receptors. ATP concentration in the extracellular space increases dramatically during tissue damage and eATP acts as a danger-associated molecular pattern (DAMP) to alert innate immune system cells for tissue repair. Similarly, eATP is present at hundreds of micromolar concentration in the tumor microenvironment (TME). However, its impact on antitumor immune response is still not well established, probably because of the complexity of the responses it induces in different cells constituting the TME. On one hand, ATP released by tumor cells concomitantly to cell death can contribute to immunogenic cell death (ICD) that is proinflammatory for the innate immune compartment and beneficial for tumor control, while on the other hand, eATP can foster immune-suppressive mechanisms within the TME, thus contributing to tumor progression and metastasis. It is well established that T-cell immunity is pivotal in limiting tumor growth and possibly eradicating neoplastic cells. T cells are limited though in their antitumor activity through different mechanisms, such as exhaustion, anergy, and senescence; the pathways resulting in these cellular outcomes are not clear. Here, we review the function of P2X7 receptor in conditioning T cell-dependent immunity against cancer.

## Introduction

Extracellular adenosine triphosphate (eATP) can transduce signal into virtually all cells through two types of plasma membrane receptors: ATP-gated ion channels termed P2X receptors (P2XRs) and G protein-coupled receptors, named P2Y receptors (P2YRs) (Burnstock, 2006). The P2XR and P2YR subfamilies consist of seven (P2XR1–7) and eight (P2YR1, 2, 4, 6, 11–14) members, respectively. Whereas the selective agonist for P2XRs is ATP, P2YRs show a rather heterogeneous selectivity for the nucleotide ligand; in fact, ATP is the selective agonist only for P2Y11R, while ADP, UDP, UTP, UDP-glucose, or UDP-galactose are agonists as well for other P2YRs. A vast repertoire of pharmacological tools has been developed to define P2 receptor functions and to be possibly used in clinical trials for various pathological conditions (Jacobson and Muller, 2016).

The P2X7R is broadly expressed in the immune system, whether innate or adaptive, and is the component of the P2XR family with a clearly defined role in a number of inflammatory and immune responses (Di Virgilio et al., 2017). In the tumor microenvironment (TME), P2X7R activity conditions the function of different cell subsets and can have opposite influences on the progression of the disease as a tumor-promoting or contrasting factor. The P2X7R monomer is a 595-aa protein that oligomerizes into a trimer to constitute the functional receptor (Surprenant et al., 1996; McCarthy et al., 2019). It is activated by relatively high extracellular concentrations of ATP (in the hundreds of micromolar range) and is characterized by dual gating. Activation of P2X7R by eATP opens the cation-selective channel within milliseconds, whereas prolonged exposure to eATP leads to dilation of a pore permeable to molecules of up to 900 Da and eventually cell death (Browne et al., 2013; Khadra et al., 2013). Whether this membrane permeabilization is due to dilation of the P2X7 channel itself (Yan et al., 2010), or the activation of non-selective pores like pannexin-1 (Pelegrin and Surprenant, 2006), gasdermin-D (Faliti et al., 2019), or anoctamin-6 (Ousingsawat et al., 2015), is probably dependent on the cell type, structural features of the plasma membrane, and/or possibly other cellular factors.

## The P2X7R in Tumor Cells

**FIGURE 1:**
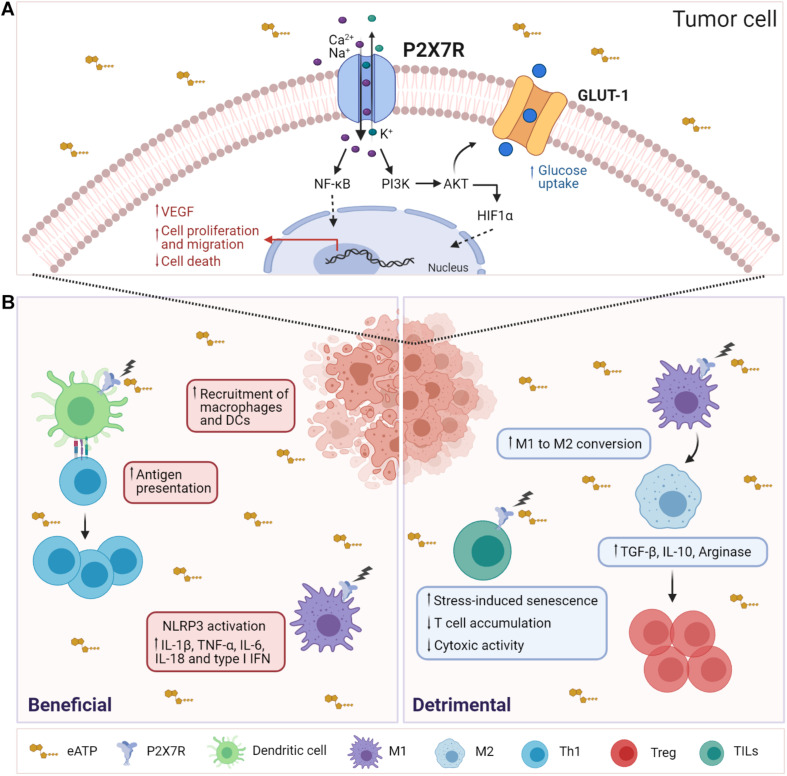
Influence of P2X7R activity on tumor and immune system cells. **(A)** In tumor cells, P2X7R stimulation by eATP results in Ca^2+^ influx, activation of MyD88/NF-κB, PI3K/Akt/mTOR signaling pathways leading to nuclear translocation of Hif1α and Nf-κB, enhancement of cell proliferation, cell migration, and VEGF secretion. Moreover, stimulation of P2X7R upregulates the glucose transporter GLUT-1, fostering the glycolytic pathway and oxidative phosphorylation that allow tumor cells to proliferate *in vitro* in the absence of serum. **(B)** (Left) In the TME, extracellular ATP is released by tumor-dying cells and may function as a DAMP (danger-associated molecular pattern), inducing the recruitment and activation of macrophages and DCs. Activation of P2X7R by eATP triggers several responses in DCs, such as maturation, migration, and antigen presentation. Moreover, K^+^ release leads to NLRP3 inflammasome activation and secretion of proinflammatory cytokines such as IL-1β, TNF-α, IL-6, IL-18, and type I IFN. (Right) P2X7R activation promotes immunosuppression by MDSCs by stimulating the release of ROS, arginase 1 (ARG1), and TGF-β1, which, in turn, leads to Tregs expansion. Moreover, P2X7R promotes the conversion of M1 macrophages to M2. P2X7R activity in TILs fosters stress-induced cellular senescence, thus limiting their expansion and cytotoxic activity.

The P2X7R is expressed in most tumor cells. For an exhaustive review of P2X7R expression in different cancers, we refer the reader to a recent publication (Lara et al., 2020). In B16 melanoma cells, low-pH that mimics features observed in solid tumors, such as hypoxia and acidosis, was shown to induce P2X7R-mediated ATP release (Hattori et al., 2012). Another study showed that P2X7R-expressing tumors were characterized by increased proliferation, reduced apoptosis, and enhanced activation of the transcription factor NFATc1. These tumors also secreted high levels of VEGF and displayed a more developed vascular network; these phenomena were inhibited by pharmacologic P2X7R blockade (Adinolfi et al., 2012). In neuroblastoma cells, P2X7R activity positively regulated the activation of the PI3K/Akt pathway, HIF1α expression, VEGF secretion, and GSK3β phosphorylation, regulating *MYCN* oncogene and glycogen accumulation. Notably, high P2X7R levels were associated with reduced survival in a cohort of neuroblastoma patients (Amoroso et al., 2015). In osteosarcoma cells, P2X7R stimulation increased glycogen storage, epithelial-to-mesenchymal transition, and stemness. The induction of PI3K/Akt/GSK3β/β-catenin and mTOR/HIF1α/VEGF signaling supported these oncogenic features in osteosarcoma cells (Zhang et al., 2019a) (Figure 1A).

In lung cancer cells, P2X7R activation by eATP could induce epithelial–mesenchymal transition (EMT), cell migration, and invasion (Cao et al., 2019). In pancreatic ductal adenocarcinoma, P2X7R activity influenced cell survival and migration *in vitro* (Giannuzzo et al., 2015). Two studies recently correlated high expression of P2X7R in colorectal cancer with poor prognosis, cancer progression, and metastasis, thus suggesting that P2X7R might be exploited as a biomarker and therapeutic target in these patients (Zhang et al., 2019b; Calik et al., 2020b). P2X7R might promote the migration and invasion of colon cancer cells by activating the STAT3 pathway (Zhang et al., 2020). Moreover, high expression of P2X7R was associated to poor survival in gastric cancer patients, suggesting that P2X7R may serve as a prognostic parameter and therapeutic target also in these patients (Calik et al., 2020a). Different studies have uncovered a role of extracellular ATP in the expansion of hematopoietic stem cells (Lemoli et al., 2004; Casati et al., 2011). P2X7R could function in the development of leukemia-initiating cells (LICs). In fact, blocking the ATP/P2X7R axis delayed leukemia development, suggesting that eATP may serve as an important niche factor in the control of LICs via P2X7R activation (He et al., 2021).

Overexpression of the full-length P2X7R in tumor cells resulted in enhanced lactate production and cell proliferation in serum-deprived culture media. This metabolic shift was defined by the upregulation of glycolytic promoters, inhibition of pyruvate dehydrogenase (PDH), enhanced phosphorylation of Akt/PKB, and expression of HIF-1α and intracellular glycogen, all metabolic modifications found in developing tumors (Amoroso et al., 2012). Recently, non-functional P2X7 (nf P2X7), which does not open to cytolytic pore, was detected in multiple cancer cell lines. High ATP concentrations induced nf P2X7 and downregulated P2X7R, leading to the hypothesis that tumor cells might avoid cytolytic pore-mediated cell death via this regulatory pathway. Notably, the 200–216 AA sequence of P2X7R was selectively exposed in nf P2X7 but not in “wild-type” P2X7R in several cancer types, and antibodies targeting this sequence have been developed as therapeutics (Gilbert et al., 2019).

The wealth of observations on P2X7R activity in different cancers suggests that P2X7R can act as an intrinsic positive regulator of tumorigenesis and metastasis generation. Therefore, the selective targeting of pro-tumorigenic signaling pathways controlled by P2X7R could provide therapeutic opportunities for oncologic patients.

## The ATP/P2X7R Axis in Tumor-Infiltrating Innate Cells

To overcome the issue of measuring the ATP concentration in tissues, Di Virgilio and collaborators developed a plasma membrane luciferase (pmeLUC) probe that is exposed on the cell surface, thus allowing the real-time measurement of extracellular ATP (Pellegatti et al., 2005). The application of this technology to cancer research allowed demonstrating that the TME is characterized by concentrations of eATP in the hundreds of micromolar range, whereas in healthy tissues, eATP is barely, if at all, detectable by pmeLUC (Pellegatti et al., 2008).

The TME can either foster an antitumor immune response or promote immunosuppression that accelerates tumor progression. In this context, deciphering the function of eATP can be difficult; in fact, the final effect of eATP would depend on its concentration, the expression of ectonucleotidases, and composition of the inflammatory infiltrate. In solid tumors, antineoplastic agents that induce immunogenic cell death (ICD), such as anthracyclines and oxaliplatin, stimulate a tumor-specific immune response that can support a successful therapeutic outcome. The release of ATP from dying cells constitutes a hallmark of ICD. ATP released during ICD functions as a “find-me” signal that attracts phagocytes to the site of ICD. Moreover, eATP-mediated purinergic stimulation of target cells results in inflammasome activation (Ghiringhelli et al., 2009; Martins et al., 2009; Aymeric et al., 2010). Notably, breast cancer patients carrying the P2X7R loss-of-function mutation E496A showed more aggressive cancer dissemination during treatment with adjuvant chemotherapy (Ghiringhelli et al., 2009). Enhancement of P2X7R-mediated activation of NLRP3 inflammasome in myeloid cells promoted the antitumor response by CD8^+^ TILs (Li et al., 2019). Accordingly, tumor-bearing P2X7R null mice showed lack of inflammatory infiltration and accelerated tumor progression (Adinolfi et al., 2015). Cyclic GMP-AMP synthase stimulator of interferon genes (cGAS-STING) signaling is involved in tumor sensing by innate immune cells (Deng et al., 2014; Woo et al., 2014). Recently, P2X7R activation was shown to facilitate transfer of tumor-derived cyclic GMP-AMP (cGAMP) to tumor-associated macrophages (TAMs), thereby enhancing STING-dependent type I IFN response and tumor immunogenicity (Zhou et al., 2020). Finally, activation of P2X7R expressed by DCs was shown to potentiate immune checkpoint blockade efficacy in mice bearing non-small cell lung cancer by enhancing IL-18 production (Douguet et al., 2021) (Figure 1B).

On the other hand, P2X7R is expressed by innate immunosuppressive cells, such as myeloid-derived suppressor cells (MDSCs), where P2X7R activity can promote radical species production, Arginase-1 accumulation, and TGF-β release, thus fostering immunosuppression (Bianchi et al., 2014). Consistent with P2X7R-mediated innate immunosuppression, P2X7R was recently shown to be highly expressed in TAMs and its deficiency inhibited the “M2-like” polarization of TAMs via downregulation of STAT6 and IRF4 phosphorylation both *in vivo* and *in vitro* (Qin et al., 2020) (Figure 1B). Altogether, these results show that P2X7R activity can condition functionally different cells of the innate immune system in the TME with opposite outcomes on tumor growth control. To exploit this knowledge in therapeutic approaches, it would be important to functionally define the innate component of tumor infiltrate on a personalized basis and/or selectively targeting a defined cell population with specific pharmacological tools.

## P2X7R-Mediated Regulation of T-Cell Adaptive Immunity

Purinergic signaling at the immune synapse is instrumental in amplifying T-cell receptor (TCR) signaling. Signal transduction by TCR and co-stimulatory molecules results in ATP release through pannexin-1 channels. In naïve T cells stimulated by cognate antigen, ATP activates P2XRs, including P2X1, P2X4, and P2X7 subtypes, in an autocrine fashion (Schenk et al., 2008; Yip et al., 2009). In human T cells, P2X1R and P2X4R segregate to the immune synapse, whereas the P2X7R remains homogeneously distributed in the plasma membrane and can sense eATP (Woehrle et al., 2010). In mouse T cell, eATP-mediated autocrine stimulation of P2XRs sustains MAPK signaling and induction of pro-inflammatory features. Hence, P2XRs antagonism can foster T-cell anergy and beneficially affect immunopathological damage in autoimmune conditions (Schenk et al., 2008). In addition, conversion of naïve CD4 T cells into immunosuppressive T regulatory cells (Tregs) by pharmacological P2XR antagonism contributes to this therapeutic outcome (Schenk et al., 2011). In human CD4 T cell, autocrine signaling by eATP via P2X7R promotes Ca^2+^ influx, NFAT nuclear translocation, and IL-2 production, suggesting that P2X7R is required for productive T-cell activation (Yip et al., 2009).

A few reports addressed the function of P2X7R in DCs during the induction of T helper cell polarization. A non-hydrolyzable ATP derivative was shown to distort DC maturation and inhibit the production of IL-12 and TNF-α; in fact, Th1 polarization of naive CD4 T cells by DCs pretreated with ATP was compromised (la Sala et al., 2001). Nevertheless, this function of P2X7R in DCs is controversial, since P2Y11R might mediate this effect through cAMP signaling (Wilkin et al., 2002; Schnurr et al., 2005). Micromolar concentrations of ATP reduce chemoattraction of Th1 and cytotoxic cells by DCs, suggesting that ATP may not only inhibit polarization of Th1 cells but also diminish the DCs/T cells interaction. Moreover, culture supernatants from ATP-treated DCs were shown to impair the migratory capacity of these cells (la Sala et al., 2002).

Some effector T-cell populations are particularly vulnerable to eATP-induced cell death via P2X7R-mediated cytolytic pore formation. For example, T follicular helper (Tfh) cells in the Peyer’s patches (PPs) of the small intestine are selectively expanded in *P2rx7^–/–^* mice because of resistance to eATP-mediated cell death (Proietti et al., 2014, 2019). P2X7R-mediated pyroptosis impairs the generation of ICOS^+^ IFN-γ-secreting Tfh cells in systemic lupus erythematosus (SLE), thus limiting immunopathology. Notably, reduced P2X7R activity characterizes circulating Tfh cells in SLE patients (Faliti et al., 2019).

In contrast, P2X7R plays a positive role in the establishment and maintenance of murine long-lived central and tissue-resident memory (Trm) CD8 T cells by supporting mitochondrial function and metabolic fitness (Borges da Silva et al., 2018). In fact, P2X7R promotes Trm cell generation by enhancing sensitivity to TGF-β (Borges da Silva et al., 2020). These results are difficult to reconcile with studies showing that P2X7R activity *in vivo* leads to a specific depletion of Trm cells (Stark et al., 2018) and intestinal T effector/memory cells (Hashimoto-Hill et al., 2017). Extracellular nucleotides released during infection and tissue damage were shown to deplete Trm cells via P2X7R unless they were stimulated via TCR that robustly downregulated P2X7R (Stark et al., 2018), as shown in small intestine Tfh cells (Proietti et al., 2014). This mechanism would allow permanence of antigen-specific over bystander T cells in a tissue niche. Because of the dual gating properties of the receptor, P2X7R could support cell autonomous maintenance of the memory T-cell pool at steady state by promoting mitochondrial fitness via its activity as ion channel; high concentrations of eATP (e.g., during inflammation) without concomitant TCR engagement would result in cytolytic pore opening and cell death, thereby selecting antigen proficient cells.

## T-Cell Function Conditioning by TME

In solid tumors, the extent of T-cell infiltration correlates with better patient prognosis. It was originally appreciated in primary cutaneous melanoma that the abundance of tumor-infiltrating lymphocytes (TILs) had a strong predictive value for increased survival (Clark et al., 1989; Clemente et al., 1996). Subsequently, the presence of intratumoral T cells was shown to correlate with improved clinical outcome in advanced ovarian carcinoma (Zhang et al., 2003). The role of adaptive immunity in controlling tumor growth was further suggested in colorectal cancers where accumulation of memory and effector memory T cells was associated with diminished dissemination and prolonged survival (Pages et al., 2005).

In the TME, the development of effective antitumor immunity is limited by the induction of T cell dysfunctional states, such as exhaustion and senescence. Exhaustion is the result of persistent antigen stimulation that provokes a gradual decrease in effector function of CD8 T cells in tumors and infections. Exhausted T cells express high levels of inhibitory molecules, including PD-1, CTLA4, Tim-3, and TIGIT, and fail to respond to TCR stimulation by cognate antigen. They are defective in killing activity and secretion of effector cytokines such as IFN-γ and TNF-α (Barber et al., 2006; Fourcade et al., 2012; He et al., 2017; Li et al., 2018). Immune checkpoint blockade modulates inhibitory pathways that affect antitumor immunity at different stages of T cell response. Anti-PD-1 antibodies were shown to unleash tumor-specific cytotoxic T cells that already reside in the TME before treatment (Tumeh et al., 2014). The rather low success rates for anti-PD-1/PD-L1 monotherapy suggests the existence of diverse adaptive immune resistance mechanisms within the TME.

Cellular senescence can be elicited by telomere shortening or erosion (termed replicative senescence) and/or “damage” signals, such as oxidative stress (termed premature senescence) (Campisi and d’Adda di Fagagna, 2007). Senescent T cells develop significant phenotypic and genotyping alterations, like a decrease in CD28 expression, cell cycle arrest, and secretion of proinflammatory and suppressive cytokines (Ye et al., 2013). In patients with head and neck cancer, CD8^+^CD28^–^ effector T cells were expanded and showed a rapid turnover. This phenomenon was normalized by tumor resection, suggesting that tumor cells promoted the generation of this “cell death-prone” subset of effector T cells (Tsukishiro et al., 2003). Senescent T cells lose their killing abilities and secretion of antitumoral cytokines, such as granzyme and IFN-γ. It was hypothesized that tumors directly induced T-cell senescence and converted effector T cells functionally into suppressor cells to achieve immune evasion (Montes et al., 2008). This hypothesis was corroborated by data suggesting that tumor cells could transfer cyclic adenosine monophosphate (cAMP) to T cells via gap junctions, resulting in T-cell senescence and immunosuppression, which could be reverted by cAMP lowering via TLR8 signaling in tumor cells (Ye et al., 2014). It is plausible to hypothesize that T-cell senescence in the TME may contribute to compromise the efficacy of multiple clinical trials of cancer immunotherapy. Thus, inhibition of T-cell senescence might constitute a strategy for restoring T-cell effector function and enhance antitumor immunity.

## P2X7 in Antitumor T-Cell Response

Tumor engraftment in P2X7R null mice showed lack of inflammatory infiltration and accelerated tumor progression, suggesting host immune system benefits from P2X7R activity for controlling tumor growth (Adinolfi et al., 2015). Conversely, pharmacological blockade of P2X7R with A740003 showed an opposite effect on tumor outcome. Immunophenotyping of tumor-infiltrating cells in P2X7R null versus A740003-treated mice uncovered a different T-cell subset composition. Robust decrease of CD8 effector T cells and increase of immunosuppressive Tregs distinguished tumors implanted in P2X7R null host. Conversely, P2X7R antagonism caused a rise of effector T cells while leaving unaltered CD8^+^ cells and Tregs numbers, suggesting that P2X7R inhibition was directly affecting effector T-cell expansion (De Marchi et al., 2019). Notably, P2X7R stimulation in tumor-specific T cells within the TME resulted in stress-induced cellular senescence that limited the expansion of tumoricidal cells. This mechanism was dependent on mitochondrial reactive oxygen species (ROS) generation and p38 MAPK-dependent upregulation of *cyclin-dependent kinase inhibitor 1A* (*Cdkn1a*, encoding for p21^*Waf*1/Cip1^) (Romagnani et al., 2020) (Figure 1B). It was previously shown in primary human T cells that inhibition of p38 MAPK could limit DNA damage and senescence-associated dysfunction independently of T cell exhaustion induced by PD1 (Lanna et al., 2014; Henson et al., 2015). More recently, inhibition of p38 MAPK activity was shown to positively influence cell expansion, differentiation, oxidative stress, and genomic stress of antitumor T cells, thereby improving the tumoricidal activity of mouse T cells and enhancing the competence of human tumor-reactive and gene-engineered T cells (Gurusamy et al., 2020). Lack of P2X7R activity in T cells correlated with a transcriptional signature associated to enhanced cytotoxic T cell response in human solid tumors. Thus, targeting of P2X7R in effector TILs might provide a unique rejuvenating signal able to sustain the tumoricidal response (Romagnani et al., 2020).

## Concluding Remarks

Signaling by eATP is diffused in all tissues; it is difficult to find a cell type that is insensitive to eATP. P2X receptors appeared very early in evolution and are present in *Dictyostelium*, *Schistosoma*, and algae (Burnstock and Verkhratsky, 2009). P2X7R is widely expressed in the different cell types constituting the TME. Dissecting the outcome of P2X7R signaling for therapeutic purposes within this complex and evolving environment represents a problematic task. Nevertheless, the knowledge acquired on P2X7R activity in various cell subsets constituting the TME might be applied to cell-targeted therapeutic approaches or for conditioning T cells in adoptive cell therapy protocols.

## Data Availability Statement

The original contributions presented in the study are included in the article/supplementary material, further inquiries can be directed to the corresponding author.

## Author Contributions

All authors listed have made a substantial, direct and intellectual contribution to the work, and approved it for publication.

## Conflict of Interest

The authors declare that the research was conducted in the absence of any commercial or financial relationships that could be construed as a potential conflict of interest.
